# A Multi-Disciplinary Approach to the Identification and Characterization of Areas of Potential Damage in the Building Materials of Ancient Monuments

**DOI:** 10.3390/s26051648

**Published:** 2026-03-05

**Authors:** Giuseppe Casula, Silvana Fais, Maria Giovanna Bianchi, Paola Ligas

**Affiliations:** 1 Istituto Nazionale di Geofisica e Vulcanologia (INGV), Sezione di Bologna, Viale Berti Pichat 6/2, 40127 Bologna, Italy; mariagiovanna.bianchi@ingv.it; 2 Department of Environmental Civil Engineering and Architecture (DICAAR), University of Cagliari, Via Marengo 2, 09123 Cagliari, Italy; sfais@unica.it (S.F.); pligas@unica.it (P.L.); 3 Consorzio Interuniversitario Nazionale per l’Ingegneria delle Georisorse (CINIGEO), Palazzo Baleani, Corso Vittorio Emanuele II 244, 00186 Roma, Italy; 4 National Research Council of Italy (CNR), Institute of Environmental Geology and Geoengineering (IGAG), Via Marengo 2, 09123 Cagliari, Italy

**Keywords:** non-invasive diagnostic techniques, terrestrial LiDAR, close-range photogrammetry, 3D models, geomatics, ultrasonic measurements, electrical resistivity, carbonate rocks, petrographic analyses

## Abstract

Today, the integrated study of historic buildings and their associated artifacts through three-dimensional modelling has become essential. Non-destructive diagnostic techniques are crucial for thorough understanding of the state of conservation of artifacts and stone construction materials used in ancient times. Therefore, it is extremely important to create digital copies that preserve the memory of the analysed forms while also allowing an understanding of the deterioration phenomena that affect historic artifacts, thus guiding restoration efforts. In this paper, the authors present the integrated application of non-destructive geomatic techniques such as terrestrial laser scanning (TLS) in synergy with close-range photogrammetry (CRP) methods, and their integration with non-destructive geophysical diagnostic methods such as ultrasonic indirect tests, ultrasonic transmission tomography, and electrical resistivity. These methods have been further enhanced by complementary petrographic analyses of the investigated building stone materials. The integrated and coordinated application of these non-destructive techniques allowed the creation of high-precision models of both the surface and interior of several artifacts from the Basilica of San Saturnino, the oldest church in Cagliari (Italy), dedicated to the city’s patron saint. Finally, this integrated study highlighted areas of deterioration of these artifacts due to atmospheric elements such as wind and rain, and anthropogenic phenomena such as atmospheric particulate matter from traffic and other manufacturing activities.

## 1. Introduction

Integrated studies in the field of non-invasive diagnostics for the characterization of stone building materials of monuments have the common target of meeting the current and future needs of society in the field of the cultural heritage. The ever-increasing demand for non-destructive technologies in cultural heritage diagnostics makes non-destructive testing (NDT) an ideal solution for the characterization and analysis of stone materials. The conservation of the built heritage requires many interventions aimed at analysing the state of health of the monuments, their preventive preservation, reconstruction, and restoration. NDT has been a key player in improving the characterization of stone materials, especially when complemented by petrographic and petrophysical analyses, which provide a comprehensive understanding of the stone’s composition, structure, and behaviour under various conditions. In fact, several studies [1,2,3,4,5] have shown that the physical, and in particular the elasto-mechanical, characteristics of rocks are significantly influenced by their mineralogical and textural properties. Therefore, when interpreting NDT data, it is essential to consider the intrinsic properties of the rocks. This ensures that the analysis is both accurate and meaningful by accounting for factors such as mineralogical composition, structure, and other inherent properties. These factors are derived from mineralogical and petrographic studies carried out on rock samples from the same quarries as the construction materials under investigation or from outcrops of the same rock type [6,7]. Knowledge of these elements is crucial for evaluating a material’s suitability for construction, its longevity, and its resistance to environmental factors.

All actions and diagnostic analyses should respect both the historical significance of the investigated monuments and the physical properties of the materials that compose the cultural heritage structures. Therefore, non-invasive techniques are the most appropriate for assessing the quality of stone building materials in monumental and historic structures. In this context, the application of various non-invasive diagnostic techniques (NDTs), such as terrestrial laser scanning (TLS), CRP, thermography, acoustic methods, electrical techniques, ground-penetrating radar and others, combined with petrographic analyses, plays a crucial role both in the preventive preservation and continuous monitoring of monumental structures. In fact, the use of such techniques is also particularly effective in controlling the effectiveness of restoration actions. The use of a single method is never totally accurate or reliable. Therefore, for an accurate diagnostic analysis, it is necessary to use and compare several methods, selected according to the nature of the building materials and their actual conditions, since each method has its own peculiarities [1,4,8,9,10,11,12,13].

Understanding the properties of stone materials and the factors influencing the evolution and distribution of degradation is essential, especially when preserving material integrity while assessing its condition.

**Figure 1:**
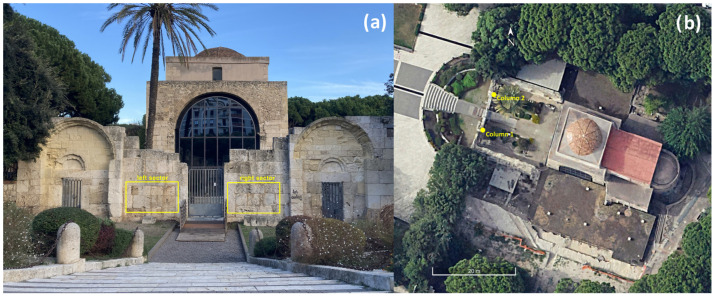
San Saturnino Basilica (Cagliari–Italy). Location of the investigated structures in the external courtyard: (**a**) in the yellow box the analysed left and right masonry sectors; (**b**) semicolumns numbered as column 1 and column 2.

In this study, we examined various architectural structures, such as masonry stone walls and semi-columns, in the Basilica of San Saturnino, the oldest early Christian monument in the historical center of Cagliari, Italy (see Figure 1). The analysis was carried out using a combination of geomatic techniques such as static TLS and CRP and geophysical methods (acoustic and electrical), complemented by petrographic analyses. The combined use of static CRP and TLS enables the generation of high-resolution 3D models or digital replicas that are accurately calibrated and textured with both the normalized intensity value of the laser beam backscattered by the surface of targeted materials and the natural color derived from digital photos [14].

Moreover, the comparison of digital 3D models resulting from the synergistic application of these two methodologies is highly effective in assessing completeness, achieving high accuracy, and recognizing details with sub-centimetric resolution [15,16]. This integration proves very useful for heritage documentation, for assessing the condition of surface materials, and for effectively planning subsequent geophysical investigations. In this study, acoustic techniques in the 24–54 kHz ultrasonic range were primarily applied in two modes: surface mode and transmission mode [17] (ISRM, 2014), both of which are highly valuable for assessing different aspects of the stone’s conditions. Among the geophysical tests used for the characterization of the stone materials, the longitudinal (P-wave) ultrasonic velocity test can be performed both in the laboratory and on-site. It is a non-destructive testing method useful and widely used in different fields of the applied research such as civil engineering [18], geomechanics [19,20], forestry [21,22], and cultural heritage [1,2,8,11,23]. The measured longitudinal velocity values and then the elastic rock properties provide key insights into the mechanical behavior of the material, making this method essential in both field practice and applied research. The 2D ultrasonic velocity maps, obtained through various data acquisition and processing techniques, effectively describe the distribution of longitudinal velocity, both on the surface of the investigated ancient walls and within the internal sections of different architectural elements, such as the semi-columns. These maps highlight both the presence of degradation or defects within the stone materials and the influence of variations in their intrinsic properties on the propagation of longitudinal ultrasonic waves.

The electrical resistivity data acquired on the surface of the old walls has allowed the mapping of the resistivity distribution on the wall surface. For porous materials such as the building’s carbonate rocks under study, electrical resistivity depends on several physical properties, including internal structure, water content, fluid composition, and porosity. Together with elastic properties, electrical resistivity can provide valuable insights into the material’s characteristics and conservation state. The integration and interpretation of multiple datasets allow for more informed, precise, and tailored interventions that address the specific needs of the building stone materials under investigation.

## 2. Materials and Methods

### 2.1. Materials

**Figure 2:**
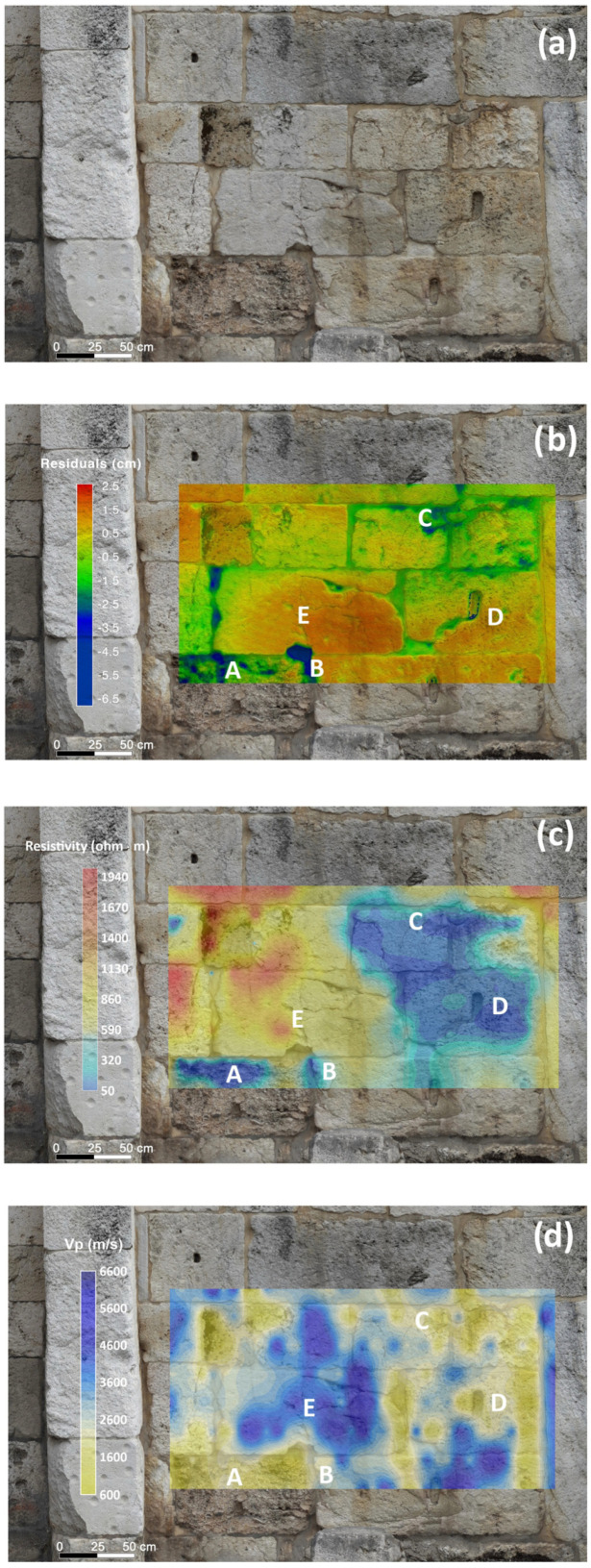
Left masonry sector in the external courtyard of the San Saturnino Basilica: comparison between geomatical and geophysical data; (**a**) frontal view of the investigated masonry deriving from the geomatic 3D model; (**b**) geometric anomalies; (**c**) resistivity map; (**d**) longitudinal velocity map.

**Figure 3:**
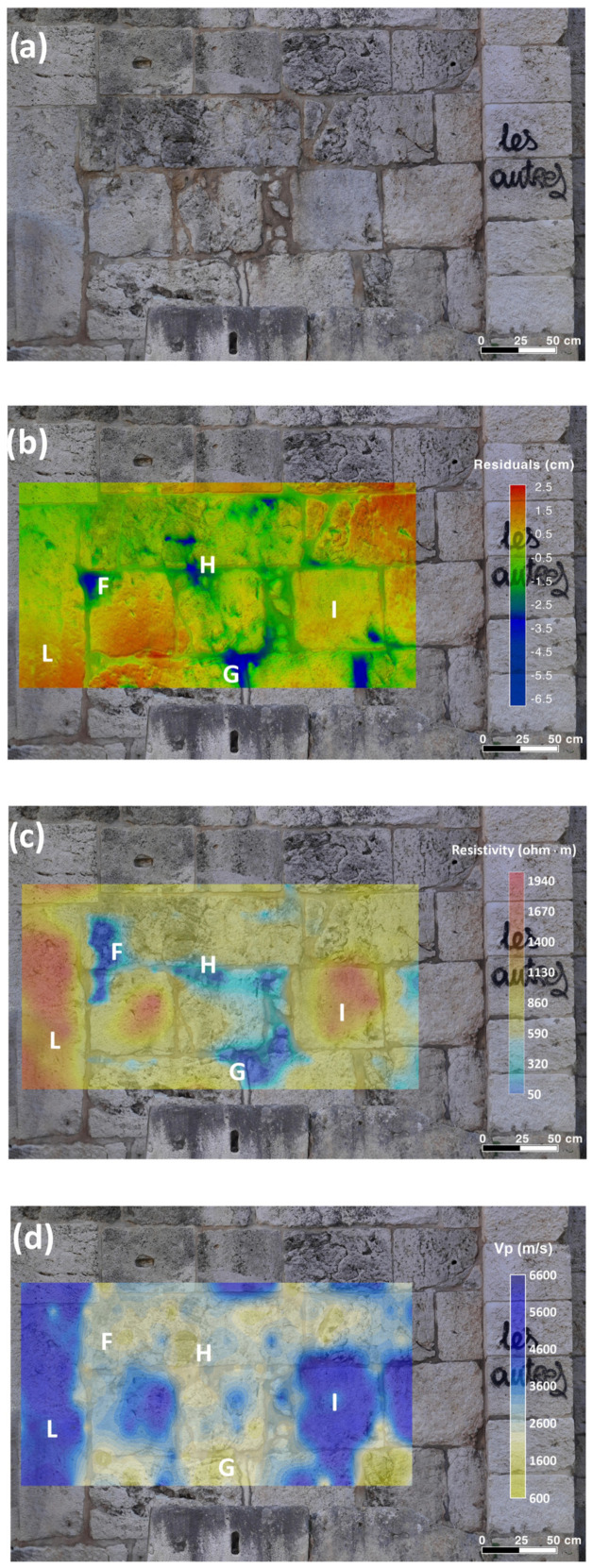
Right masonry sector in the external courtyard of the San Saturnino Basilica: comparison between geomatical and geophysical data; (**a**) frontal view of the investigated masonry deriving from the geomatic 3D model; (**b**) geometric anomalies; (**c**) resistivity map; (**d**) longitudinal velocity map.

**Figure 4:**
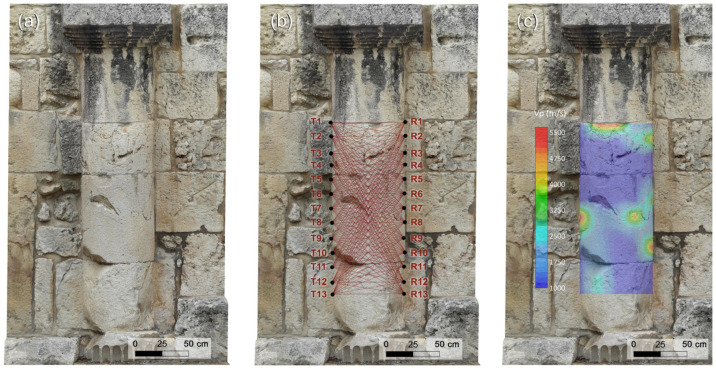
2D tomography on column 1 in the courtyard of the San Saturnino Basilica: (**a**) digital photogrammetric model; (**b**) acquisition scheme of the 2D tomography; (**c**) result of the 2D tomography superimposed on the digital photogrammetric model.

**Figure 5:**
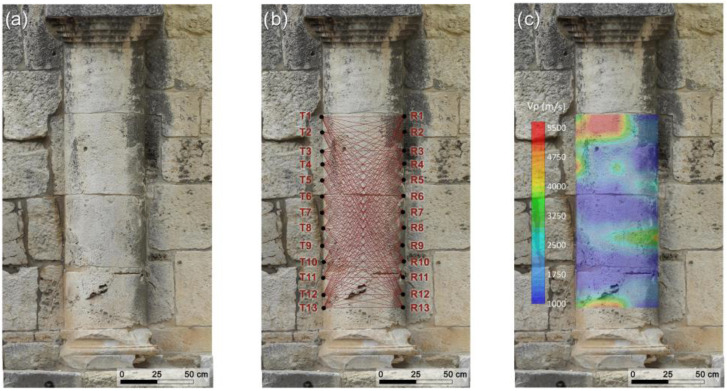
2D tomography on column 2 in the courtyard of the San Saturnino Basilica: (**a**) digital photogrammetric model; (**b**) acquisition scheme of the 2D tomography; (**c**) result of the 2D tomography superimposed on the digital photogrammetric model.

The Basilica of San Saturnino is made up of a variety of building materials (limestones, sandstones, marbles, volcanic rocks and others), which is related to the complex history and transformation of the monument over the centuries. Nevertheless, a large number of building materials belong to the carbonate Miocene stratigraphic sequence named “Calcari di Cagliari” formation [24,25]. The ashlars of the walls of the external courtyard of the present main façade (Figure 2a and Figure 3a) and the building materials of the studied semi-columns (Figure 4a and Figure 5a) in the XI-XII Cent. AD courtyard [26] prevalently belong to the same geological formation. As is well known, widespread outcrops in the neighbouring areas of the town of Cagliari are made up of these rocks [24,27]. The choice of building materials by the builders of the past, as well as their artistic taste and technical requirements, was driven by their accessibility and/or availability. Therefore, this choice was closely linked to the local geology of the historical/archaeological sites. Furthermore, this was one of the most important aspects towards the containment of construction costs.

The “Calcari di Cagliari” date back to the Tortonian–lower Messinian and are made up of three different types of carbonate rocks named respectively from top to bottom Pietra Forte or Calcari di Bonaria, Tramezzario and Pietra Cantone. These carbonate rocks are characterised by different textural features depending on the history and instability of the depositional basin and in relation to the paleobathymetry in which they were formed. The hypothesized bathymetry is from 60 to 80 m for Pietra Cantone, not exceeding 40 m for Tramezzario and at about 30 m for Pietra Forte [28]. These lithotypes have been studied and characterised by their mineralogical, petrographic, physical/petrophysical and elasto-mechanical features [6,7,11,23,29,30,31], and their pathologies of decay have also been analysed with particular attention paid to the Mediterranean climate [4,32]. The data of the above-mentioned studies have been crucial for both interpreting and validating the indirect measurements conducted in the present study.

From preliminary visual observations of the investigated structures, we were able to make a preliminary distinction, excluding the attribution of the building materials to Pietra Cantone (Figure 2a, Figure 3a, Figure 4a and Figure 5a). We need to consider that Pietra Cantone is generally easily recognised on visual inspection since macroscopically it appears typically yellowish in colour and with the intrinsic features distinctive of a poorly cemented fine-grained carbonate rock, as reflected in the conspicuous characteristic forms of decay such as alveolization, which is also named honeycomb weathering [7,29]. Considering the high alterability of Pietra Cantone, Columbu et al. (2017) [30] have proposed consolidating the more degraded materials using protective chemical treatments. Pietra Cantone bioclastic limestone is characterised by a micritic texture rich in foraminifera with small amounts of iron oxides, quartz, feldspars and biotite [7]. It is classified as mudstone–wackestone [33] with an 80–86% CaCO_3_ content [29], high values of effective porosity, up to 40%, and low values of permeability, up to 11 mD [7]. The generally high tortuosity of the pore system, up to 23 [7], is in accordance with the small-sized particles (micrite matrix) and the diffuse primary microporosity of the mud matrix. Those textural features determine the high hygroscopicity and vulnerability to degradation of this lithotype [2].

In its macroscopic appearance Tramezzario presents the characteristics of a white-coloured bioclastic compact carbonate rock. However, when this rock is cut into ashlars and installed in building structures, it displays degradation forms such as black crusts and/or appears weakly cemented with high secondary porosities (i.e., fissures and cracks). In the most severe stages of the decay, Tramezzario can completely pulverize into a carbonate mud. Previous petrographic studies [7,29] have highlighted a grain-supported limestone rich in bioclasts mostly made of Lithothamnium algae and minor molluscs in a sparry calcite cement. This limestone is classified as grainstone–packstone [33] with CaCO_3_ values in the 87–98% range [29]. Tramezzario is frequently characterized by well-interconnected macroporosity and microporosity with high values of effective porosity, up to 41%, permeability up to about 1170 mD and low tortuosity of the pore network, up to 4 [7]. The well-interconnected pore network offers an easy passage to the fluids (high permeability and low tortuosity), thus favoring decay processes in the Tramezzario.

Upon visual observation the Pietra Forte is white-cream-colored and generally appears to be a very compact and tenacious carbonate rock. In many cases the red-brown patinas due to the oxidation of iron minerals produce detachment and exfoliation of the brittle materials in the installed ashlars. Petrographic analyses have highlighted a biohermal–biostroma limestone in which the original components, mainly Lithothamnium algae, embody bioclasts cemented by sparry calcite [7,29]. The Pietra Forte is classified as a boundstone [33] and like the Tramezzario it is almost entirely made up of CaCO_3_ (88–98%) [29]. Effective porosity, up to 10%, and permeability, up to 0.16 mD, are generally low [7]. Tortuosity of the pore network of the Pietra Forte is quite low, up to 7, and is related to the intrinsic textural features of this rock [7]. In synthesis, even considering that this lithotype is less susceptible to degradation, in some cases the presence of fractures and dissolution phenomena of calcite forms “karsts” with caves and fissures, significantly increases the permeability of this lithotype.

### 2.2. Geomatic Data

Two Terrestrial LIDAR surveys and four digital photogrammetric experiments were planned and conducted on two sections of the ancient masonry walls located in the external courtyard of the San Saturnino Basilica archaeological complex in the city of Cagliari.

The TLS surveys were carried out mainly on 4 November 2023 and 11 November 2024 by, with the aid of a levelled geodetic tripod at different station points, first installing a Leica HDS-6200 PS (phase scanner) laser scanner and then also a Leica BLK360 laser scanner with TOF + WFD (time of fly + wave form digitalisation) technologies. For every campaign, six high-density point clouds were acquired in a few hours and at different station points all around the walls to be modelled.

During later digital photogrammetric campaigns, four individual surveys were carried out on four separate days: 5, 7, and 8 November 2023, and 11 November 2024. For these surveys, a commercial single-lens digital camera, specifically, a Canon PowerShot 730 HS CMOS with a resolution of 20.3 megapixels, was used to acquire several hundred high-quality two-dimensional images of the masonry walls under investigation. The photographs were systematically taken around the target structure at close range (1–2 m) from various station points, which were evenly spaced at an average interval of 30 cm, thereby ensuring a 70–80% overlap between images. All photographs were taken, to the greatest extent possible, under uniform lighting conditions. Additionally, simple high-contrast scale bars were strategically positioned on, adjacent to, or in proximity of the surveyed targets.

### 2.3. Geomatic Methods

#### 2.3.1. Terrestrial LIDAR

In the Terrestrial LIDAR technology, a laser beam of known frequency and wavelength (generally in the near infrared) is projected onto the surface of the object to be surveyed by means of a system of oscillating and rotating mirrors with adjustable pitch on an extremely precise grid, while the corresponding backscattered radiation is received and acquired by the laser sensor. Knowing the speed of the laser beam, the sensor is able to determine the position of the target points in cylindrical coordinates, which are then converted into orthogonal Cartesian coordinates that provide the position of the pixel and the intensity of the reflected light as well as the natural colours in an RGB scale in the event of the laser being equipped with an optical camera. The corresponding array of point coordinates, intensity of light and natural colours of the materials represent a point cloud. There are essentially three types of laser scanners currently in use for civil engineering and architectural purposes: time-of-flight (TOF) scanners calculate the distance based on the time the laser beam takes to travel from the instrument to the targeted object and back; phase shift scanners are based on the computation of the phase shift between the signal emitted and that reflected by the target; and finally the latest generation hybrid systems, for example systems that use wave form digitizer (WFD) technology (as the Leica BLK360), and combine time-of-flight and phase shift measurement technology. In the latter sensors the distance is determined by measuring the time between a start and a stop pulse digitized from the received signal. The WFD system continuously analyses, digitizes, and accumulates the waveform of all reflected signals to accurately identify and extract the start and stop pulses [34].

#### 2.3.2. CRP

In the CRP based on the Structure from Motion (SfM) methodology, arrays of good-quality 2D images are collected with high-precision optical cameras all around the studied object at predetermined known distances with a 70 to 80% degree of overlap, mounted on tripods, and equipped with high dynamic range (HDR) technology, which is effective in recovering from exposure error. Images are post-processed with algorithms derived from computer graphics, to convert photos to 3D HR models as unstructured unified point clouds and meshes texturized with the natural colours of the images [35,36,37].

#### 2.3.3. Data Processing

CRP—At the beginning the image orientation procedure is performed. This is done by suitably implemented algorithms of aerial triangulation (AT) applied together in bundle block adjustment (BBA). Thousands of feature points are detected and across images converted into tie points. During the image orientation procedure, the position and photogrammetric calibration of the cameras is performed to determine the camera’s interior orientation parameter values, including the parameters of distortion of the camera lens.

In particular, the following calibration parameters are determined/adjusted: the focal length measured in pixels; the coordinates of the principal point (i.e., the coordinates of the lens optical axis interception with the sensor plane in pixels); the affinity and non-orthogonality (skew) coefficients (in pixels); and the radial and tangential distortion dimensionless coefficients (Agisoft Metashape software Vs 2.0 LLC. St. Petersburg, Russia) [38,39].

At the end of the fine image alignment procedure a sparse point cloud is detected and visualised together with the camera positions array. The sparse point cloud-selected stereo pairs are then used to compute the depth maps, based on the estimated camera positions and images (dense stereo matching) and a dense point cloud is then built [40].

The processing procedure follows on with camera calibration and colours calibration, and with the dense cloud colouring procedure using the images. A very dense 3D mesh is then computed using the highest resolution achievable. This mesh, texturized with the natural colours of the images used, is then utilised to recompute a high-density cloud starting an iterative process that ends when the 3D model is implemented in the best way, and the highest accuracy is reached (generally less than 1 mm).

The resulting high-resolution 3D point cloud can be manually filtered and scaled using calibration bars suitably implemented and inserted in the scene. The obtained 3D model can be exported in E57 or PLY formats (namely formats for LIDAR data exchange) to be made available as input for the software for point cloud processing like JRC 3D Reconstructor^®^ Vs 4.1.2 (Gexcel, Brescia, Italy) and the CloudCompare Vs 2.13.2 free software.

The terrestrial LIDAR data processing is performed with the aid of JRC-3D Reconstructor^®^ Vs 4.1.2 and CloudCompare Vs 2.13.2.

The procedure starts with a format conversion and input of point clouds. The point clouds are then pre-processed with manual and automatic filtering to eliminate unwanted data and to compute the pixel normals. A point clouds draft and fine registration is then performed with ICP (Iterative Closest Point) and similar bundle adjustment algorithms [11,41,42]. Once the point clouds acquired in different station points are finely registered, they are aggregated in a unified, registered, filtered and unstructured point cloud representing the HR 3D model of the studied artifacts. Finally, polygonal meshing and geometrical anomalies are computed and used for comparison with other non-destructive diagnostic methods like ultrasonic ones. In fact, to account for the studied artifacts’ surface anomalies like crack, fissures, voids and wrinkled areas in the surfaces of the blocks, we need to compare the 3D models with graphic primitives with regular surfaces like planes in the case of walls, while cylinders are fitted to columns [43]. The residuals are computed adopting the meshed primitives as reference. The result of the geophysical methods can also be compared with the intensity of the TLS light.

Moreover, CRP based on the Structure from Motion (SfM) methodology is characterised by high productivity but lacks scale and coordinates [35,36]. Conversely the synergistic application of terrestrial LIDAR together with CRP makes it possible to verify the scale factor (i.e., scale and coordinates) of the 3D models computed with the photogrammetry technique. Indeed, the scale factors are adjusted by comparing the 3D models derived by the two techniques using the algorithms for bundle adjustment suitably implemented in the CloudCompare Vs 2.13.2 and Reconstructor Vs 4.1.2 software packages, thus providing a highly accurate means of measurement.

### 2.4. Geophysical Measurements

Based on our understanding of the textural characteristics of the investigated stone materials, and utilizing 3D reconstructions obtained through TLS and CRP, a geophysical survey was planned and carried out. The geomatic and geophysical investigations focused on some significant historic structures of the San Saturnino Basilica, including historic masonry and semi-columns. Ultrasonic testing with indirect modality [17] (ISRM, 2007–2014) and electrical resistivity measurements were carried out on the selected masonry sectors of the external courtyard of the San Saturnino Basilica to generate detailed maps of longitudinal velocity and resistivity distributions. These results provide important insights into the conservation state of masonry elements exposed to weathering and other atmospheric agents. The relationship between resistivity and longitudinal velocity in rocks is mainly influenced by the mineral composition, porosity, and fluid content of the rock. The relationship between resistivity and longitudinal velocity (P-wave velocity) in rocks is not straightforward or direct, but their comparison still holds significant value in non-invasive diagnostic analysis, particularly when assessing the conservation state of stone materials.

Additionally, 2D ultrasonic transmission tomography [17] (ISRM, 2007–2014) was employed to identify the size and location of potential textural heterogeneities and internal defects within the semi-columns of the above courtyard. Variations in longitudinal velocity observed in different areas of the stone materials reflect changes in their elastic properties, which may result from textural heterogeneities, internal cracking, material degradation, or structural detachments.

#### 2.4.1. Resistivity Measurements

The electrical resistivity of stone materials can vary significantly, influenced by factors such as mineral composition, porosity, salinity and resistivity of pore fluid, water content, and clay content [44]. Consequently, it can serve as a useful indicator of the materials’ susceptibility to degradation. However, the interpretation of the resistivity distribution for a detailed diagnosis of the degradation of the investigated stone materials is a difficult task considering that this parameter is controlled by many factors, especially porosity. Stones with higher porosity tend to retain more moisture, which generally lowers resistivity. However, the relationship between porosity and resistivity is not straightforward. The connectivity between the pores is also crucial, as it determines how easily moisture can flow through the stone and impact its overall resistivity. The connectivity is also strictly related to the size and arrangement of pores, pore-throats and pore-to-pore connections [6,7]. Poor connectivity between pores prevents uniform moisture distribution, resulting in localised variations in resistivity. The tortuosity of the pore network also greatly affects the interconnectivity of the pore system. The high tortuosity that characterizes the pore network favours resistance to current flow, leading to an increase in resistivity.

In our study, resistivity measurements were carried out along eight parallel profiles, spaced 15 cm apart in the vertical direction on the surface of the investigated sectors of the historic masonry walls. The measuring points for the geo-electrical resistivity measurements were spaced at a constant interval of 7.5 cm in the x-direction along each profile. This acquisition geometry enables high-resolution two-dimensional resistivity mapping of the masonry wall surface. Based on the spacing of the current electrodes, the investigated depth ranged consistently from 5.0 cm to 7.5 cm. This depth is appropriate for evaluating the near-surface condition of the masonry, where deterioration processes, such as moisture ingress and material loss, typically originate.

The measurements were performed using the Resipod device (Proceq, Schwerzenbach, Switzerland). The Resipod is an integrated Wenner quadrupole probe designed to measure the electrical resistivity of various materials through a completely non-destructive testing method.

The Wenner configuration offers particularly good resolution for surface investigations and ensures a high signal-to-noise ratio [45]. The resistivity measurements involve two pairs of electrodes: the current electrodes 15 cm apart that inject a known electric current into the material, and the potential electrodes that measure the resulting potential difference, which is used to calculate the resistivity of the stone material.

Resistivity values were contoured to visualise their spatial distribution on the investigated masonry sectors, aiming to gather valuable information on their conservation state which would complement the ultrasonic data.

#### 2.4.2. Ultrasonic Measurements

Non-destructive techniques based on the propagation of elastic waves are well-established and widely used in assessing the condition of stone building materials. These methods are based on the principle that wave propagation velocity is influenced by the material’s density, elasto-mechanical properties and petrographic characteristics [7,10,12,23,46,47]. Furthermore, the presence of internal defects such as cracks, voids, textural heterogeneities or other structural anomalies can significantly affect wave propagation.

In this study, ultrasonic measurements were performed in accordance with the [17] ISRM (2007–2014) guidelines, using a portable Ultrasonic Non-Destructive Digital Indicating Tester (PUNDIT Lab Plus, Proceq–Switzerland) paired with a Fluke 96B oscilloscope for digital signal acquisition, display, and processing. The PUNDIT device measures the propagation time of ultrasonic pulses over a range of 0.1–9999.0 μs with a precision of ±1 μs.

Silicone snug sheets have been recognized as one of the most effective coupling agents for ultrasonic measurements on porous rocks [7,11]. This type of coupling agent offers significant advantages by enhancing the transmissibility of the ultrasonic signal and compensating for surface irregularities at the interface. Moreover, silicone snug sheets prevent the coupling material from penetrating the porous carbonate materials, thereby avoiding potential interference with the ultrasonic test. Piezoelectric transducers with a frequency range of 24–82 kHz were used depending on the material’s condition and the specific objectives of the investigation for each monumental element. The frequencies adopted for the different tests were selected as an acceptable compromise between resolving power and tolerable attenuation.

In general, higher frequencies provide greater resolution and are more sensitive to small defects or surface features. However, they are also more strongly absorbed and scattered by the material. As a result, their penetration depth is limited compared with lower frequencies, which can reach deeper layers with lower resolution. Ultrasonic signals can be processed to analyse the different frequency components. Techniques such as spectral analysis help to separate and interpret high- and low-frequency signals, aiding in the identification of specific elasto-mechanical properties of building stone materials.

#### 2.4.3. Ultrasonic Test on the Masonry Walls

Based on previous in situ ultrasonic tests, a frequency of 82 kHz was chosen for application on the external surface of the investigated historic masonry walls (Figure 1). This frequency is characterized by shorter wavelengths, which provide higher-resolution imaging and allow for the detection of finer details and shallow features within the material.

The tests on the historic masonry walls were performed with an accuracy of ±1% in indirect transmission mode [17] (ISRM, 2007–2014), meaning that the transmitter and receiver were placed on the same surface of the wall. This approach is suitable for obtaining ultrasonic longitudinal velocity data from the shallow layers of the walls and for comparing them with geomatic and geoelectric ones. The ultrasonic method is based on the transmission of elastic waves between pairs of measurement stations (transmitter and receiver) positioned at accurately known distances on the wall surface. The ultrasonic measurements were carried out on the investigated masonry sectors along eight parallel profiles, spaced 15 cm apart from each other in the vertical direction. The transmitter–receiver distance for the in situ measurements was set at 15 cm. The ultrasonic transducers were moved at regular 7.5 cm intervals along the x-direction for each profile in order to increase the measurement density and allow for a more detailed analysis of the longitudinal velocity distribution. Great attention was given to ensuring that all measurements were performed at the same height across all profiles. At each measurement, the transit times of the ultrasonic signals from transmitter to receiver were obtained by precisely detecting the first arrival of the received longitudinal ultrasonic signal on the Fluke oscilloscope interfaced with a laptop for data acquisition and processing. To enhance the signal-to-noise ratio, five signals were stacked at each measurement point. Stacking effectively suppressed randomly generated noise and facilitated the identification of the ultrasonic first arrivals used to determine the transit times. The propagation velocity at each observation point was then computed from the known transmitter–receiver spacing and the measured transit times. It should be noted, however, that the obtained pulse velocity represents an apparent velocity, as its calculation assumes a minimum linear distance between the transmitter and receiver stations, whereas in reality, the wave may follow a more complex propagation path within the material. Nevertheless, the presence of defects and heterogeneities can be effectively detected, allowing the method to yield valuable insights into the internal condition of the building material.

The measurement grid covered the same area previously investigated through the resistivity measurements described above. The calculated longitudinal velocities were then used to generate 2D contour maps, visually representing the distribution of longitudinal wave velocities within the superficial layers of the investigated masonry sectors. This approach aimed to identify damages, degradation zones and textural heterogeneities by analyzing the spatial velocity variations and comparing them with geomatic and geoelectric data. This comparison highlights the spatial correlation between the measured ultrasonic data and the physical features of the wall surface.

#### 2.4.4. Ultrasonic Test on the Semi-Columns

Starting from the knowledge of the textural characteristics of the carbonate materials constituting the investigated semi-columns, and utilizing 3D models obtained through geomatic techniques (CRP and TLS), a two-dimensional (2D) ultrasonic tomographic test was designed and carried out to analyze the internal elasto-mechanical properties of the building materials along vertical cross-sections within the semi-columns. The ultrasonic tomography of transmission was performed by analyzing the first arrivals of the ultrasonic signals, which were reliably attributed to longitudinal wave (P-wave) propagation.

The morphology (shallow features) of the investigated semi-columns was analyzed by the 3D digital photogrammetric models (Figure 4a and Figure 5a) to design an optimal ultrasonic survey and provide a very good coverage of the examined cross-sections.

The ultrasonic measurements were carried out using the above-mentioned PUNDIT Lab Plus (Proceq–Switzerland) coupled with a Fluke 96B digital oscilloscope to monitor the received waveforms in real-time. The measurements were performed with an accuracy of ±1% by the direct transmission mode [17] (ISRM 2007–2014), in which the transmitter and receiver were positioned on opposite sides of the investigated cross-sections (Figure 4b and Figure 5b). The velocity of the longitudinal wave between the transmitter–receiver path can be estimated by considering their distance divided by the transit time. Defects such as voids, cracks, or degraded zones along the propagation path reduce the pulse velocity, whereas zones exhibiting better elastic properties than the surrounding material lead to an increase in velocity. The low ultrasonic frequency of 24 kHz was found to be effective for detecting potential internal defects (heterogeneities) within the semi-columns. Preliminary tests conducted with transducers of various frequencies showed that ultrasonic wave attenuation at 24 kHz was negligible relative to the thickness of the semi-columns examined in this study. Therefore, the ultrasonic signals are expected to propagate through the full thickness of the semi-columns without significant energy loss. The station points were spaced at 10 cm intervals along a longitudinal extent of 120 cm for each cross-section.

The 2D tomography conducted on the vertical cross-sections of the two investigated semi-columns comprised 338 measurements for each, resulting from the combination of 13 source points and 13 receiver points positioned on opposite sides of the cross-sections. Each station was alternately used as both a transmitter and a receiver. Then, the investigated sectors were traversed by a dense network of intersecting ideal rays at various inclinations (Figure 4b and Figure 5b). Using the 3D digital photogrammetric models, the positions of the station points, their coordinates within a fixed reference system, and the transmitter–receiver distances were accurately determined. Subsequently, the travel times of the longitudinal ultrasonic signals along the source–receiver paths were recorded, together with the corresponding position and orientation of each ray within the investigated cross-sections. To enhance the signal to noise ratio (SNR) of the ultrasonic signals and facilitate first-break recognition in the waveform, four successive signals for the same propagation path were stacked. First-break times were manually picked and quality-controlled to ensure consistency and eliminate erroneous travel-time picks. The precise identification of the P-wave first arrival time in the received ultrasonic signal is crucial for ensuring the accuracy and reliability of tomographic reconstructions. The travel times corresponding to each path shown in Figure 4b and Figure 5b were measured and subsequently modeled by considering curved ray paths and employing the well-established Simultaneous Iterative Reconstruction Technique (SIRT) inversion algorithm [48,49]. This technique is based on iterative approximations aimed at refining an arbitrary initial parameter distribution, defined by the starting velocity model. To derive a realistic non-arbitrary starting velocity model for the SIRT inversion of travel-time data and to obtain a reliable 2D representation of the ultrasonic longitudinal wave velocity distribution within the investigated structures, a methodology based on the cross-correlation function [50] was employed. This function acted as a constraint in the SIRT tomographic inversion, enabling the integration of prior information about the investigated cross-sections. The principal steps of the procedure have been reported in previous studies [1,6,7]. This procedure enabled the creation of 2D maps of the ultrasonic longitudinal velocity distribution within the semi-columns, highlighting regions with distinct elastic properties. The 2D distribution of longitudinal velocities within the investigated cross-sections was visualized using Surfer v.30.2.240 (Golden Software), which provides a precise and reliable two-dimensional representation.

## 3. Results and Discussion

Integrating complementary data obtained from geomatic and geophysical techniques offers a powerful diagnostic approach to assess the conservation state of the building materials of valuable historical and architectural structures. Geomatic techniques, such as TLS and CRP, provide high-resolution spatial and morphological data, enabling precise metric documentation and spatial analysis, which in turn leads to accurate modeling of surface morphology and shallow potential deformation.

The geophysical techniques used in this study, such as resistivity and ultrasonic techniques, supply subsurface information revealing internal decay, presence of defects, textural heterogeneities and moisture distribution. Knowledge of the petrographic characteristics of the investigated building materials helps the analysis and interpretation of the responses of the geomatic and geophysical data. A summary of the key finding obtained in this study for each applied non-invasive technique is provided in Table 1.

### 3.1. Geomatics

As detailed in the section dedicated to the description of the methods, the application of TLS and photogrammetric techniques to the ancient masonry walls in the external courtyard of the archaeological complex of the San Saturnino Basilica resulted in the generation of high-resolution 3D models of the study areas to the left and right of the central entrance (see Figure 2a and Figure 3a). These 3D models are essentially the registered, filtered and aggregated point clouds, which serve as digital replicas of the masonry study sectors and are textured using natural colours obtained from photographs captured with the Canon PowerShot 730 optical camera (Canon Inc., Ōita, Japan). Owing to the specific characteristics of the survey, it was possible to achieve models with high metric accuracy, as the scale factor of the photogrammetric models was further implemented and refined with the utmost precision utilizing the 3D models derived from the available TLS surveys.

Figure 2b and Figure 3b represent the high-resolution 3D models alongside the geometric anomalies, superimposed on the models textured with natural colours. These anomalies were calculated as residuals with respect to the meshes of the planes fitted to the rectangular sectors under investigation.

The anomalies are visualized using a “rainbow” styled colour scale: raised areas are indicated in red hues, while areas lower than the fitted planes are in blue, corresponding to depressed zones of the wall.

The patterns of these anomalies exhibit interesting similarities with the results obtained from the application of other non-destructive techniques, such as the ultrasonic and electrical resistivity one discussed later. To verify the accuracy of the 3D models shown in Figure 2b and Figure 3b, with regard to the left and right sectors of the ancient masonry wall, the cloud-to-cloud absolute distances between the photogrammetric model and the terrestrial lidar-derived 3D model were calculated using the dedicated function integrated in the Cloud Compare analysis package (C2C), [51] as well as the Multiscale Model to Model Cloud Comparison (M3C2) function [52] from the same software, followed by the application of Gaussian statistics to the corresponding residuals.

The C2C method is the most straightforward and efficient approach for directly comparing point clouds in 3D, as it does not require gridding, meshing, or the computation of surface normals [51,52]. Essentially, it calculates the distance between two points clouds acquired at different epochs—commonly referred to as the interpoint distance.

In this approach, the first point cloud serves as reference, and for each point in the second cloud, the nearest corresponding point in the reference dataset is identified. In its simplest implementation, surface change is then derived from the distance between each of these point pairs.

In contrast, the M3C2 method incorporates several key features. It directly processes point clouds without the need for gridding or meshing and determines the local distance between two points clouds along the surface normal direction, thereby capturing three-dimensional variations in surface orientation. Furthermore, it computes a confidence interval for each distance measurement by accounting for point cloud roughness and registration uncertainty. Together, these characteristics enable the method to achieve greater accuracy than the C2C approach [52,53].

**Figure 6:**
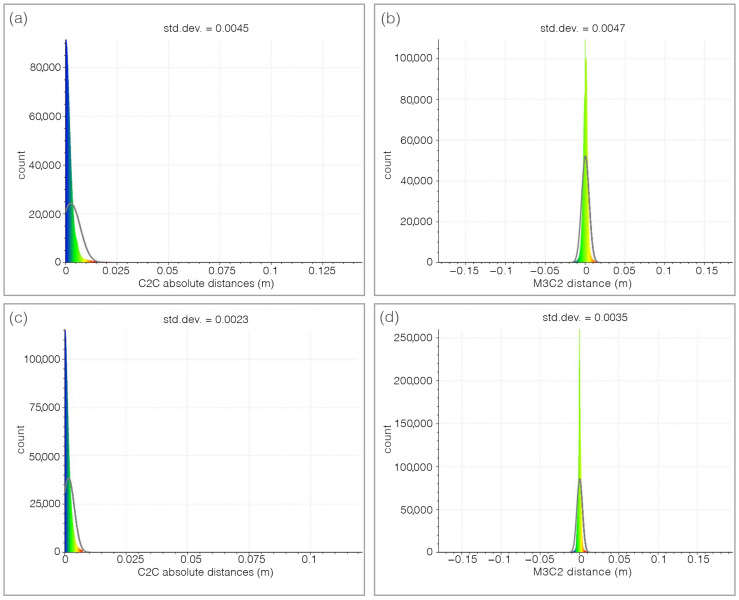
Histograms illustrating the Gaussian statistical analysis of the cloud-to-cloud (C2C) distances and the Multiscale Model to Model Cloud Comparison (M3C2) for the high-resolution 3D models of the investigated sectors of the ancient perimeter wall, located in the external courtyard of the San Saturnino Basilica archaeological complex. The calculated standard deviations for these measurements range from 2.3 to 4.7 mm.

The results of the two latter operations are consistent, as can be seen in the histograms presented in Figure 6. Specifically, the 3D model of the right part of the wall shows a standard deviation of the absolute C2C distances of 4.5 mm (Figure 6a), while the standard deviation of the M3C2 distances is 4.7 mm (Figure 6b). Regarding the three-dimensional model of the left part of the ancient perimeter wall, the standard deviation obtained using the C2C technique amounts to 2.3 mm (Figure 6c), while the one relating to the test performed with the M3C2 technique is 3.5 mm (Figure 6d). Looking at Figure 6, it is evident that the standard deviation values of the distances between the point clouds are close to approximately half a centimeter. This outcome is attributable to the presence of geometric anomalies within the analysed sections of the wall, characterized by typical raised and depressed zones on the wall surface that can reach extreme values in some cases exceeding ±2.5 cm (refer to Figure 2b and Figure 3b).

Despite the differences between the two methods used to assess the accuracy of the 3D models of the right and left sides of the external courtyard wall, no appreciable discrepancies were observed. The only notable difference is that, as shown in Figure 6, the standard deviations calculated using the M3C2 method are slightly higher than those obtained with the C2C method. Moreover, no systematic variations were detected between the 3D models produced using TLS and photogrammetry. On the contrary, clear similarities emerge between the two models, although the CRP model exhibits a higher resolution and its scale factor requires improvement with the aid of the TLS 3D model.

### 3.2. Geophysical Measurements

The results of the geomatic and geophysical survey for the investigated sectors of the masonry walls of the main entrance to the external courtyard of the San Saturnino Basilica are shown in Figure 2a–d and Figure 3a–d. The results of the tomographic test superimposed on the digital photogrammetric models on the columns 1 and 2 of the interior of the courtyard are shown in Figure 4a–c and Figure 5a–c respectively.

#### 3.2.1. Masonry Walls

In both sectors of the investigated masonry walls, the resistivity maps (Figure 2c and Figure 3c) show values ranging approximately from 50 to 1900 Ωm. The pronounced spatial variability of resistivity reflects the combined influence of moisture distribution across both sectors of the masonry and the textural characteristics of the Pietra Forte and Tramezzario ashlars, which constitute the main lithological components of the investigated masonry walls.

Zones characterized by resistivity values below approximately 400 Ωm are interpreted as zones with elevated moisture content and greater porosity, conditions that enhance the susceptibility of the stone to decay mechanisms such as salt crystallization, microfracturing, and biological colonization. Conversely, higher resistivity values (>800 Ωm) generally correspond to less porous and less altered materials, indicative of better preservation. A comparative analysis of the geometrical anomalies and electrical resistivity maps reveals that negative geometrical anomalies often correspond with zones of low resistivity. Notably, negative geometrical residuals, i.e., zones **A**, **B** and **C** correspond to low-resistivity zones **A**, **B** and **C** (Figure 2b,c) in the left sector of the masonry. Similarly, in the right sector, negative residuals **F**, **G** and **H** coincide with low-resistivity zones **F**, **G** and **H** (Figure 3b,c). This relationship suggests a common underlying cause, such as increased moisture content or material degradation highlighting areas particularly susceptible to weathering and decay. Such zones are considered critical, as they often represent the initial loci from which degradation processes originate and subsequently propagate through the surrounding material. In some other cases, positive geometrical residuals are associated with higher resistivity values. For example, see zone **E** in Figure 2b,c and zones **I** and **L** in Figure 3b,c, which suggest the presence of denser, less altered material. In a few cases, positive geometrical anomalies correspond to low-resistivity zones (e.g., anomaly **D** in Figure 2b,c). This condition could be justified by the fact that some carbonate building materials of the investigated masonry are characterized by high porosity and interconnected pore systems, which facilitate fluid infiltration and enhance alteration processes. In the urban and nearshore setting of the Basilica of San Saturnino, marine aerosols, humidity, and rainwater with pollutants introduce salts that penetrate the pores. Repeated dissolution–crystallisation cycles then generate stress on the pore walls, causing material swelling and deterioration. The swelling likely explains the positive geometric anomalies associated with low-resistivity and low-velocity zones.

The ultrasonic longitudinal velocity maps (Figure 2d and Figure 3d) obtained by the indirect measurement modality [17] (ISRM 2007–2014), provide a clear representation of the spatial variability of the elastic conditions of the shallow building materials. In both sectors of the investigated walls, the longitudinal velocity maps display values ranging from approximately 600 m/s to 6000 m/s, revealing strong elastic heterogeneity despite the limited compositional variation in the carbonate building materials (Pietra Forte and Tramezzario). This evidence suggests that the elastic response of the building materials is more strongly governed by textural characteristics, such as grain size, packing, and pore structure than by differences in mineralogical composition. Furthermore, as previously mentioned, voids, fissures, discontinuities, or alteration features reduce the propagation velocity of the longitudinal elastic waves, as they attenuate the ultrasonic signal by absorbing part of its energy.

Although the two carbonate facies, Pietra Forte and Tramezzario, which constitute almost the entire wall structures, are difficult to distinguish visually, by comparison with previous laboratory data [7,29], high-velocity zones (Vp > 4500 m/s, indicated in blue on the velocity map) can be correlated with the presence of Pietra Forte.

In some cases, the comparison between geometrical anomalies, resistivity and longitudinal velocity maps shows a good correspondence, despite the fact that the different methods investigate different depths of the near surface materials. For example, the area indicated as zone **E** in the maps of the left sector (Figure 2b–d) presents a clear correspondence among the different datasets: the positive geometrical anomalies (+2.0 cm) correspond to an area of relatively high resistivity (greater than 800 Ωm) and high velocity (>4500 m/s). Based on these results, zone **E** could be identified as Pietra Forte and can be considered to be in a good state of preservation. A similar observation can be made for zones **I** and **L** when analyzing the maps of the right sector of the masonry wall. In these zones, positive geometrical anomalies are associated with relative high resistivity and high velocity values (Figure 3b–d).

The critical zones discussed above, shown as zones **A**, **B**, **C** (left sector) and zones **F**, **H**, **G** (right sector) in the resistivity maps, associated with negative geometrical anomalies and indicative of material loss or morphological irregularities, are also characterized by low ultrasonic velocities (1500–2000 m/s), confirming their degraded and critical conditions (Figure 2b–d and Figure 3b–d).

#### 3.2.2. Semi-Columns

The two semi-columns in the courtyard of the Basilica of San Saturnino, referred to as column 1 and column 2 in Figure 1, are made up of carbonate rocks which, based on our previous studies [1,2,7,29] and geological knowledge, were identified as Pietra Forte. Preliminary visual inspections reveal shallow alteration phenomena, including fracturing, material detachment caused by physical weathering, and the formation of black crusts (Figure 4a and Figure 5a). No signs of previous restoration were perceived. The presence of fractures and karst features, such as cavities and fissures, significantly increases the permeability of this lithotype, promoting the degradation processes and consequently affecting its elastic behaviour. Previous ultrasonic laboratory tests indicate that intact Pietra Forte generally has a wave velocity in the range 5000–7000 m/s, while deterioration can lower these values to roughly 1000–2000 m/s [2,7,29].

**Figure 7:**
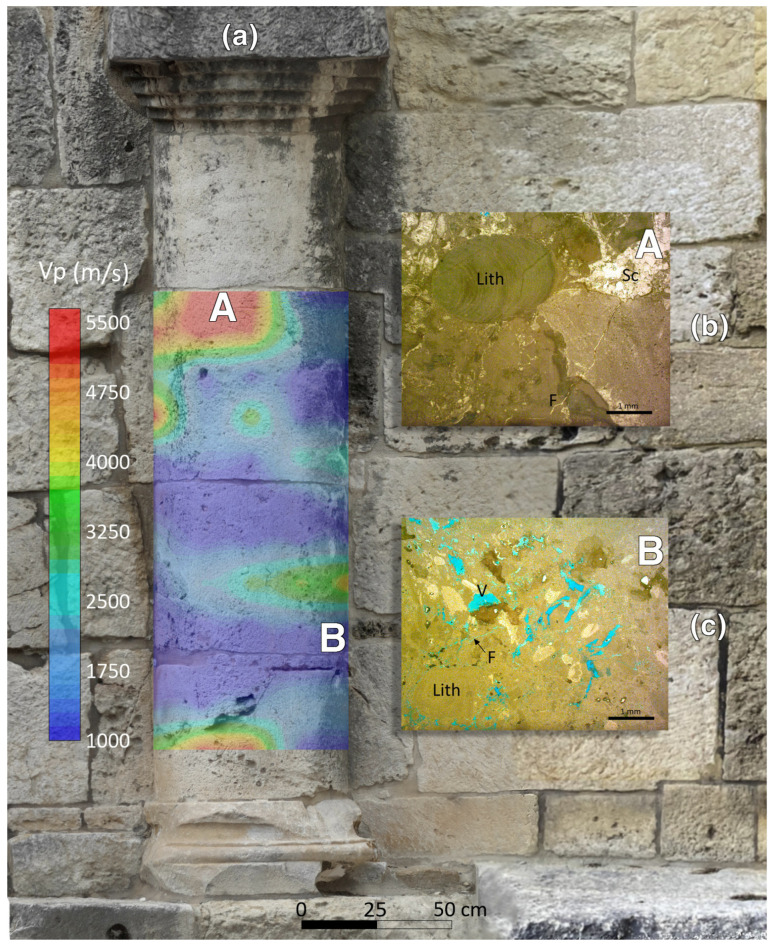
Integrated interpretation of the results of the 2D tomography and thin-section analyses on column 2: (**a**) result of the 2D tomography superimposed on the digital photogrammetric model; (**b**) microscopic textural features of Pietra Forte with higher degree of cementation—zone A. Lithothamnium algae (Lith) and fractures (F) filled with sparry calcite (Sc). OM plane polarized light. Sample treated by blue dye epoxy resin; (**c**) microscopic textural features of Pietra Forte with advanced degradation—zone B. Type of porosity is well highlighted by blue dye: fracture (F), vug (V). OM plane polarized light.

Low-frequency ultrasonic longitudinal-wave transmission tomography, performed using the SIRT inversion algorithm, enabled a detailed reconstruction of the internal velocity field along longitudinal sections of the semi-columns 1 and 2. The resulting 2D velocity distributions, superimposed on the photogrammetric digital models, are shown in Figure 4c and Figure 5c. This integration allows for precise correlation between the internal velocity variations and the actual geometry of the columns, improving the localization of material heterogeneities, defects, and areas of deterioration, and providing essential information for conservation or intervention strategies. Analysis of the tomographic longitudinal cross-sections reveals a wide velocity range from 1000 m/s to 5500 m/s. This variability reflects the textural characteristics of the carbonate materials, which undergo progressive deterioration when exposed to environmental agents. The resulting increase in heterogeneity and porosity leads to a marked reduction in elastic and mechanical properties. The predominantly low velocities (blue zones, below 2000 m/s) observed across both semi-columns indicate advanced and widespread degradation of the carbonate materials. Considering that the blue zones show velocity values <50% of the reference value (6000–7000 m/s) as results from velocity measurements on sound rocks sampled from the same quarries or from near outcropping rocks [7,29], it can be inferred that the stone materials in these zones are affected by high microcracking, voids, marked loss of cohesion, and presence of macropores. In these zones important textural variations in the carbonate building materials occur, especially present in different types of porosity and the presence of discontinuities among bioclasts (see for example Figure 7a,c—zone B). In order to ensure the long-term preservation of these architectural elements, the materials in low-velocity zones (blue zones) must undergo intensive consolidation interventions or, where deterioration is advanced, selective replacement of the damaged material. The above diagnosis supports the decision to carry out repair or reinforcement. These zones may be prioritized for the restoration actions, grouting, or structural support.

In a few zones of the column’s cross-sections, higher velocities were observed V < 20–30% (orange-red zones in Figure 4c and Figure 5c) with respect to the reference value (6000–7000 m/s), indicating better elastic–mechanical conditions. The elastic characteristics are improved due to greater compactness and a higher degree of cementation due to a biogenic texture. This condition greatly reduces the porosity and discontinuities inside the carbonate material (see for example Figure 7a,b—zone A). These elastic–mechanical improvements suggest a lower susceptibility to deformation and cracking with direct implications for restoration strategies. These areas may not require intensive reinforcement interventions, as the reduced porosity and lower internal discontinuity enhance the material’s ability to withstand mechanical stress and degradation.

## 4. Conclusions

Given the increasingly urgent need nowadays to create high-resolution 3D models of historical artifacts (such as the ancient perimeter masonry walls and columns located in the external courtyard of the San Saturnino Basilica archaeological complex in Cagliari), it is equally essential to model both the surfaces and the internal structures of the objects under study. To this end, it is crucial to synergistically employ advanced geomatic techniques, including terrestrial laser scanning and digital photogrammetry, in conjunction with geophysical methods. Moreover, for a more precise characterization of the stone materials comprising these monuments, their state of preservation, and the ongoing processes of deterioration, it is also essential to conduct comprehensive petrographic investigations aimed at thoroughly examining the textural and mineralogical characteristics of the rocks used as building materials. This knowledge improves the integrated interpretation of geomatic and geophysical data supporting better-informed conservation strategies.

In this work, all these methodologies have been integrated to establish a comprehensive investigative procedure, which has enabled the creation of high-resolution 3D models of the Romanesque masonry and the architectural elements under examination. These digital twins provide detailed insights into both the intrinsic characteristics of the construction materials and their state of conservation. Although the high productivity of geomatic techniques, such as terrestrial LiDAR and close-range photogrammetry, is limited to monitoring only the surfaces of materials, it nevertheless enables the creation of highly accurate and metrically precise three-dimensional digital models of the artifacts under study. Therefore, geomatics-based referencing enables the precise repetition of non-invasive diagnostic surveys on the same structures, maintaining spatial correspondence across successive measurement campaigns and ensuring the long-term comparability of results. Nevertheless, these methods alone are insufficient for conducting a thorough diagnostic analysis of the materials themselves. This limitation can be addressed by synergistically combining complementary geophysical techniques, such as the ultrasonic and resistivity techniques employed in this study and others.

The comparative analysis of data acquired through different techniques therefore provides valuable diagnostic insights for identifying vulnerable areas and understanding the spatial evolution of deterioration processes.

The workflow applied in this study is adaptable to different and complex monumental structures, while remaining sensitive to their individual characteristics. A decision-making framework is recommended to guide interventions. The cost of the proposed non-invasive diagnostic approach depends on the complexity of the monumental structure, as more articulated geometries and heterogeneous materials require longer survey times and more extensive data processing. By providing reliable, non-destructive insights into material properties, this approach supports informed conservation strategies and sound decision-making in heritage management, taking into account the condition and significance of the heritage asset, risks of deterioration, potential impact of interventions, and long-term sustainability.

A well-planned restoration strategy allows for the efficient allocation of financial resources, thereby enhancing the overall effectiveness of the intervention.

Ultimately, the approach used in this study facilitates the visualization and communication of results for engineers, conservators, and other stakeholders.

## Figures and Tables

**Table 1:** Key features of non-invasive diagnostic techniques applied in this study.

Method ^1^	Depth of Investigation	Information	Limitations
TLS	Surface/shallow subsurface anomalies	High-resolution 3D models; position and coordinates; laser intensity values	High operational costs; sometimes difficult to use in constrained environments.
CRP	Surface/shallow subsurface anomalies	High-resolution 3D models; RGB natural-color texturing	Strongly affected by lighting conditions; limited by close-range acquisition requirements.
ER	Subsurface anomalies	Indicator of moisture and porosity distribution in the building materials Susceptibility to degradation	Without knowledge of compositional and textural features, resistivity data can easily be misinterpreted.
UT	Subsurface in-depth anomalies	Elasto-mechanical behaviour Detection of shallow and in-depth defects Inspection of the entire investigated volume	Data acquisition, processing and interpretation need skilled operators. The rough surface morphology of the investigated material can cause loss of signal

^1^ TLS (terrestrial laser scanner), CRP (close-range photogrammetry), ER (electric resistivity); UT (ultrasonics).

## Data Availability

The data presented in this study are available on request from the corresponding author.

## References

[1] Casula G., Fais S., Cuccuru F., Bianchi M.G., Ligas P., Sitzia A. (2021). Decay Detection in an Ancient Column with Combined Close-Range Photogrammetry (CRP) and Ultrasonic Tomography. Minerals.

[2] Casula G., Fais S., Cuccuru F., Bianchi M.G., Ligas P. (2023). Diagnostic Process of an Ancient Colonnade Using 3D High-Resolution Models with Non-Invasive Multi Techniques. Sensors.

[3] Zhou Z., Liu N., Sun Y., Wang Z., Al Attabi K. (2025). Effect of Textural and Physical Properties of the Carbonate Rocks on Dynamic Elastic Modulus: Application of Statistical and Intelligent Methods. Phys. Chem. Earth Parts A/B/C.

[4] Sitzia F., Lisci C., Mirão J. (2021). Building Pathology and Environment: Weathering and Decay of Stone Construction Materials Subjected to a Csa Mediterranean Climate Laboratory Simulation. Constr. Build. Mater..

[5] Pang M., Ba J., Carcione J.M., Balcewicz M., Siegert M., Tang G., Saenger E.H. (2025). Structural and Elastic Properties of Carbonate Rocks with Different Pore Types Based on Digital and Theoretical Rock Physics. J. Geophys. Res. Solid Earth.

[6] Fais S., Cuccuru F., Casula G., Bianchi M.G., Ligas P. (2019). Characterization of Rock Samples by A High-Resolution Multi-Technique Non-Invasive Approach. Minerals.

[7] Casula G., Fais S., Cuccuru F., Bianchi M.G., Ligas P. (2024). An Integrated Petrographic, Geomatic and Geophysical Approach for the Characterization of the Carbonate Rocks of the Calcari Di Cagliari Formation. Minerals.

[8] Centauro I., Vitale J.G., Calandra S., Salvatici T., Natali C., Coppola M., Intrieri E., Garzonio C.A. (2022). A Multidisciplinary Methodology for Technological Knowledge, Characterization and Diagnostics: Sandstone Facades in Florentine Architectural Heritage. Appl. Sci..

[9] Dionísio A., Martinho E., Grangeia C., Almeida F. (2013). Examples of the Use of Non-Invasive Techniques for the Evaluation of Stone Decay in Portugal. Key Eng. Mater..

[10] Fais S., Cuccuru F., Ligas P., Casula G., Bianchi M.G. (2017). Integrated Ultrasonic, Laser Scanning and Petrographical Characterisation of Carbonate Building Materials on an Architectural Structure of a Historic Building. Bull. Eng. Geol. Environ..

[11] Fais S., Casula G., Cuccuru F., Ligas P., Bianchi M.G. (2018). An Innovative Methodology for the Non-Destructive Diagnosis of Architectural Elements of Ancient Historical Buildings. Sci. Rep..

[12] Salvatici T., Calandra S., Centauro I., Pecchioni E., Intrieri E., Garzonio C.A. (2020). Monitoring and Evaluation of Sandstone Decay Adopting Non-Destructive Techniques: On-Site Application on Building Stones. Heritage.

[13] Zielińska M., Rucka M. (2018). Non-Destructive Assessment of Masonry Pillars Using Ultrasonic Tomography. Materials.

[14] Sánchez-Aparicio L.J., Blanco-García F.L.D., Mencías-Carrizosa D., Villanueva-Llauradó P., Aira-Zunzunegui J.R., Sanz-Arauz D., Pierdicca R., Pinilla-Melo J., Garcia-Gago J. (2023). Detection of Damage in Heritage Constructions Based on 3D Point Clouds. A Systematic Review. J. Build. Eng..

[15] Conti A., Pagliaricci G., Bonora V., Tucci G. (2024). A Comparison between Terrestrial Laser Scanning and Hand-Held Mobile Mapping for the Documentation of Built Heritage. Int. Arch. Photogramm. Remote Sens. Spat. Inf. Sci..

[16] Mustafin M., Nasrullah M., Abboud M. (2024). 3D Modeling of Sidon Sea Castle Utilizing Terrestrial Laser Scanner Combined with Photogrammetry. Int. J. Geoinformatics.

[17] Ulusay R., ISRM (2014). Upgraded ISRM Suggested Method for Determining Sound Velocity by Ultrasonic Pulse Transmission Technique. Testing and Monitoring: 2007–2014.

[18] Vasanelli E., Colangiuli D., Calia A., Sileo M., Aiello M.A. (2015). Ultrasonic Pulse Velocity for the Evaluation of Physical and Mechanical Properties of a Highly Porous Building Limestone. Ultrasonics.

[19] Christaras B. (2003). P-Wave Velocity and Quality of Building Materials. Proceedings of the International Symposium on Industrial Minerals and Building Stones.

[20] Kılıç Ö. (2006). The Influence of High Temperatures on Limestone P-Wave Velocity and Schmidt Hammer Strength. Int. J. Rock Mech. Min. Sci..

[21] Puxeddu M., Cuccuru F., Fais S., Casula G., Bianchi M.G. (2021). 3D Imaging of CRP and Ultrasonic Tomography to Detect Decay in a Living Adult Holm Oak (*Quercus ilex* L.) in Sardinia (Italy). Appl. Sci..

[22] Taskhiri M.S., Hafezi M.H., Harle R., Williams D., Kundu T., Turner P. (2020). Ultrasonic and Thermal Testing to Non-Destructively Identify Internal Defects in Plantation Eucalypts. Comput. Electron. Agric..

[23] Christaras B., Cuccuru F., Fais S., Papanikolaou H., Lollino G., Giordan D., Marunteanu C., Christaras B., Yoshinori I., Margottini C. (2015). Application of Non Destructive Ultrasonic Techniques for the Analysis of the Conservation Status of Building Materials in Monumental Structures. Engineering Geology for Society and Territory—Volume 8.

[24] Gandolfi R., Porcu A. (1967). Contributo Alla Conoscenza Delle Microfacies Mioceniche Delle Colline Di Cagliari (Sardegna). Riv. Ital. Paleont..

[25] Barca S., Melis E., Annino E., Cincotti F., Ulzega A., Orrù P., Pintus C. (2005). Note Illustrative della Carta Geologica d’Italia alla scala 1:50.000, F. 557 Cagliari. Serv. Geol. Italia—ISPRA.

[26] Sitzia F. (2021). The San Saturnino Basilica (Cagliari, Italy): An Up-Close Investigation about the Archaeological Stratigraphy of Mortars from the Roman to the Middle Ages. Heritage.

[27] Cherchi A. (1974). Appunti Biostratigrafici Sul Miocene Della Sardegna (Italia). Mem. BRGM.

[28] Leone F., Pontillo C., Spano C., Carmignani L., Sassi F.P. (1992). Benthic Paleocommunities of the Middle–Upper Miocene Lithostratigraphic Units from the Cagliari Hills (Southern Sardinia, Italy). Contribution to the Geology of Italy with Special Regard to the Paleozoic Basement. A Volume Dedicated to Tommaso Cocozza IGCP Project N°276, Newsletter 5.

[29] Cuccuru F., Fais S., Ligas P. (2014). Dynamic Elastic Characterization of Carbonate Rocks Used as Building Materials in the Historical City Centre of Cagliari (Italy). Q. J. Eng. Geol. Hydrogeol..

[30] Columbu S., Lisci C., Sitzia F., Buccellato G. (2017). Physical–Mechanical Consolidation and Protection of Miocenic Limestone Used on Mediterranean Historical Monuments: The Case Study of Pietra Cantone (Southern Sardinia, Italy). Environ. Earth Sci..

[31] Columbu S., Antonelli F., Sitzia F. (2018). Origin of Roman Worked Stones from St. Saturno Christian Basilica (South Sardinia, Italy). Mediterr. Archaeol. Archaeom..

[32] Sitzia F., Lisci C., Mirão J. (2021). Accelerate Ageing on Building Stone Materials by Simulating Daily, Seasonal Thermo-Hygrometric Conditions and Solar Radiation of Csa Mediterranean Climate. Constr. Build. Mater..

[33] Dunham R.J. (1962). Classification of Carbonate Rocks According to Depositional Textures. Am. Assoc. Pet. Geol..

[34] Maar H., Zogg H.-M. (2014). Leica WFD—Wave Form Digitizer Technology White Paper.

[35] Snavely N., Seitz S.M., Szeliski R. (2008). Modeling the World from Internet Photo Collections. Int. J. Comput. Vis..

[36] Westoby M.J., Brasington J., Glasser N.F., Hambrey M.J., Reynolds J.M. (2012). ‘Structure-from-Motion’ Photogrammetry: A Low-Cost, Effective Tool for Geoscience Applications. Geomorphology.

[37] Iglhaut J., Cabo C., Puliti S., Piermattei L., O’Connor J., Rosette J. (2019). Structure from Motion Photogrammetry in Forestry: A Review. Curr. For. Rep..

[38] Angelini A., Cozzolino M., Gabrielli R., Gentile V., Mauriello P. (2023). Three-Dimensional Modeling and Non-Invasive Diagnosis of a Huge and Complex Heritage Building: The Patriarchal Basilica of Santa Maria Assunta in Aquileia (Udine, Italy). Remote Sens..

[39] Semyonov D. Agisoft Metashape User Manual Standard Edition, Version 2.0.

[40] Rahman A.D.M., Cahyono A.B. (2023). Analysis of 3-D Building Modeling Using Photogrammetric Software: Agisoft Metashape and Micmac. IOP Conf. Ser. Earth Environ. Sci..

[41] Besl P.J., McKay N.D. (1992). Method for Registration of 3-D Shapes. Proceedings of the Sensor Fusion IV: Control Paradigms and Data Structures.

[42] He Y., Liang B., Yang J., Li S., He J. (2017). An Iterative Closest Points Algorithm for Registration of 3D Laser Scanner Point Clouds with Geometric Features. Sensors.

[43] Guerra M.G., Galantucci R.A. (2020). Standard Quantification and Measurement of Damages through Features Characterization of Surface Imperfections on 3D Models: An Application on Architectural Heritages. 13th CIRP Conference on Intelligent Computation in Manufacturing Engineering, CIRP ICME ’19. Procedia CIRP.

[44] Kahraman S., Öğretici E. (2024). Prediction of Physico-Mechanical Rock Characteristics from Electrical Resistivity Tests. J. S. Afr. Inst. Min. Metall..

[45] Schueremans L., Van Rickstal F., Venderickx K., Van Gemert D. (2003). Evaluation of Masonry Consolidation by Geo-Electrical Relative Difference Resistivity Mapping. Mater. Struct..

[46] Benavente D., Galiana-Merino J.J., Pla C., Martinez-Martinez J., Crespo-Jimenez D. (2020). Automatic Detection and Characterisation of the First P- and S-Wave Pulse in Rocks Using Ultrasonic Transmission Method. Eng. Geol..

[47] Costamagna E., Santana Quintero M., Bianchini N., Mendes N., Lourenço P.B., Su S., Paik Y.M., Min A. (2020). Advanced Non-Destructive Techniques for the Diagnosis of Historic Buildings: The Loka-Hteik-Pan Temple in Bagan. J. Cult. Herit..

[48] Trampert J., Leveque J.-J. (1990). Simultaneous Iterative Reconstruction Technique: Physical Interpretation Based on the Generalized Least Squares Solution. J. Geophys. Res..

[49] Phillips W.S., Fehler M.C. (1991). Traveltime Tomography: A Comparison of Popular Methods. Geophysics.

[50] Fais S., Casula G. (2010). Application of Acoustic Techniques in the Evaluation of Heterogeneous Building Materials. NDT E Int..

[51] Girardeau-Montaut D., Roux M., Marc R., Thibault G. (2005). Change Detection on Points Cloud Data Acquired with a Ground Laser Scanner. Proceedings of the XXXVI/3-W19/V/3 Workshop “Laser scanning 2005”.

[52] Lague D., Brodu N., Leroux J. (2013). Accurate 3D Comparison of Complex Topography with Terrestrial Laser Scanner: Application to the Rangitikei Canyon (N-Z). ISPRS J. Photogramm. Remote Sens..

[53] Diaz V., Van Oosterom P., Meijers M., Verbree E., Ahmed N., Van Lankveld T., Kolbe T.H., Donaubauer A., Beil C. (2024). Comparison of Cloud-to-Cloud Distance Calculation Methods—Is the Most Complex Always the Most Suitable?. Recent Advances in 3D Geoinformation Science.

